# Integrated Transcriptomic and Metabolomic Analysis Reveals Nitrogen-Mediated Delay of Premature Leaf Senescence in Red Raspberry Leaves

**DOI:** 10.3390/plants14152388

**Published:** 2025-08-02

**Authors:** Qiang Huo, Feiyang Chang, Peng Jia, Ziqian Fu, Jiaqi Zhao, Yiwen Gao, Haoan Luan, Ying Wang, Qinglong Dong, Guohui Qi, Xuemei Zhang

**Affiliations:** College of Forestry, Hebei Agricultural University, Baoding 071001, China; hq2289609818@163.com (Q.H.); cafiya33@163.com (F.C.); jiapeng@hebau.edu.cn (P.J.); whiyfm@163.com (Z.F.); a1361831627@163.com (J.Z.); gaoyiwen2023@126.com (Y.G.); luanhaoan@163.com (H.L.); wangyingwy5@126.com (Y.W.); bdqgh@sina.com (G.Q.)

**Keywords:** leaf senescence, metabolome, red raspberry, transcriptome

## Abstract

The premature senescence of red raspberry leaves severely affects plant growth. In this study, the double-season red raspberry cultivar ‘Polka’ was used, with N_150_ (0.10 g N·kg^−1^) selected as the treatment group (T150) and N_0_ (0 g N·kg^−1^) set as the control (CK). This study systematically investigated the mechanism of premature senescence in red raspberry leaves under different nitrogen application levels by measuring physiological parameters and conducting a combined multi-omics analysis of transcriptomics and metabolomics. Results showed that T150 plants had 8.34 cm greater height and 1.45 cm greater ground diameter than CK. The chlorophyll, carotenoid, soluble protein, and sugar contents in all leaf parts of T150 were significantly higher than those in CK, whereas soluble starch contents were lower. Malondialdehyde (MDA) content and superoxide anion (O_2_^−^) generation rate in the lower leaves of T150 were significantly lower than those in CK. Superoxide sismutase (SOD) and peroxidase (POD) activities in the middle and lower functional leaves of T150 were higher than in CK, while catalase (CAT) activity was lower. Transcriptomic analysis identified 4350 significantly differentially expressed genes, including 2062 upregulated and 2288 downregulated genes. Metabolomic analysis identified 135 differential metabolites, out of which 60 were upregulated and 75 were downregulated. Integrated transcriptomic and metabolomic analysis showed enrichment in the phenylpropanoid biosynthesis (ko00940) and flavonoid biosynthesis (ko00941) pathways, with the former acting as an upstream pathway of the latter. A premature senescence pathway was established, and two key metabolites were identified: chlorogenic acid content decreased, and naringenin chalcone content increased in early senescent leaves, suggesting their pivotal roles in the early senescence of red raspberry leaves. Modulating chlorogenic acid and naringenin chalcone levels could delay premature senescence. Optimizing fertilization strategies may thus reduce senescence risk and enhance the productivity, profitability, and sustainability of the red raspberry industry.

## 1. Introduction

Raspberry (*Rubus idaeus* L.), a perennial deciduous shrub of the genus Rubus in the Rosaceae family, is referred to as raspberry in Chinese herbal medicine. Red raspberry is rich in ellagic acid, vitamins, anthocyanins, and other bioactive compounds. These help reduce the risk of chronic metabolic diseases through various mechanisms, including anti-cancer, antioxidant, and cardioprotective effects [1,2,3,4]. Thus, it holds significant medicinal and health value. The fruit can be processed into freeze-dried products, jam, fruit wine, and seed oil [5,6,7,8] and is particularly popular in developed countries. Red raspberry is also tolerant to low temperatures and insect pests, making it a rapidly expanding economic forest crop [9,10]. Current research mainly focuses on the functional components of its fruit and its physiological responses to abiotic stress, while studies on the physiological and molecular mechanisms of premature leaf senescence remain limited.

Senescence is a critical phase in the plant life cycle. In rice, early-senescence mutants exhibit significantly reduced plant height and fruiting rates [11,12]. As leaves senesce, soluble protein content declines while total amino acid levels rise [13]. Some researchers propose that chlorophyll degradation is the hallmark of senescence, with declining chlorophyll levels causing leaves to turn from green to yellow—an evident sign of premature senescence [14]. Studies show a positive correlation between chlorophyll content and plant vitality. In living cells, chlorophyll resides in the thylakoid membrane as part of chlorophyll–protein complexes. During senescence, the number and structure of grana alter, and the outer chloroplast membrane along with the thylakoid system membrane disintegrate [15]. Moreover, excessive accumulation of O_2_^−^ is closely associated with reduced photosynthetic pigment content. These reactive oxygen species (ROS) specifically degrade chlorophyll a and lower the chlorophyll a/b ratio [16].

During leaf senescence, nucleic acid content decreases significantly, especially rRNA in chloroplasts and mitochondria, while mRNA levels of genes encoding proteases, nucleases, and related enzymes increase. Enzymatic activity leads to the degradation of chlorophyll, pigment-binding proteins, lipids, starch, and other cellular components [15]. Abscisic acid (ABA), a plant growth inhibitor, induces the expression of *senescence-associated genes* (*SAGs*) when applied exogenously [17,18]. Ethylene, a gaseous hormone, promotes senescence by activating chloroplast-degradation-related genes through its response factor *Ethylene Insensitive3* (*EIN3*) [19]. *Myelocytomatosis oncogene 2* (*MYC2*), the core transcription factor in the jasmonic acid (JA) signaling pathway, suppresses *Catalase Gene* (*CAT*) expression, enhances H_2_O_2_ accumulation, and thereby accelerates leaf senescence [20]. When salicylic acid (SA) and ethylene act synergistically, *EIN3* and *NPR1* (*Nonexpressor of pathogenesis-related genes 1*), the central regulator of SA signaling, jointly upregulate senescence-related genes such as *ORE1* (*Oresara 1*) and *SAG29 (Senescence-Associated Gene 29)*, further promoting leaf senescence [21]. However, at low concentrations, SA can delay methyl jasmonate (MeJA)-induced senescence by enhancing antioxidant capacity via nitric oxide signaling [22].

Plant growth, development, and responses to environmental stimuli involve complex regulatory networks. Single-omics approaches often fail to capture the full regulatory landscape. In contrast, multi-omics integration overcomes data limitations by linking molecular processes across different biological levels. Transcriptomics reveals differentially expressed genes, while metabolomics identifies the end products of gene expression. Together, they provide a more comprehensive bioinformatics framework, bridging causality and outcomes [23]. Currently, integrated transcriptomic and metabolomic analyses are widely applied in studies of environmental responses, fruit quality, and color variation [24,25,26]. For instance, Mei et al. (2021) used both approaches to investigate flower color differences between a rare mutant and the wild-type *Camellia* variety, revealing gene expression and metabolic pathway changes underlying the phenotype [27]. Wang et al. (2021) demonstrated that high nitrogen fertilizer suppresses carbohydrate accumulation and flavonoid biosynthesis in apples through transcriptomic, proteomic, and metabolomic analyses [28]. Similarly, Mehta et al. (2024) combined metabolomics, transcriptomics, and gene co-expression analyses to reconstruct the complete biosynthetic pathway of Amaryllidaceae alkaloids in daffodils [29]. Han et al. (2024) employed integrated transcriptional and metabolic analyses to elucidate the role of the *TAR1* (*HIV-1 TAR RNA*-*binding protein 1*) gene in trichome development and artemisinin biosynthesis in *Artemisia annua* [30]. Such combined omics analyses have become increasingly prevalent in research on major crops.

‘Polka’ has excellent storage and harvesting characteristics, a prolonged harvest period, and a high survival rate. Observations of its growth and assessments of fruit quality revealed strong tree vigor and vigorous branch development. Its fruit size and quality surpass those of ‘Heritage’, a variety widely cultivated in China. Currently, ‘Polka’ is being extensively promoted and planted across China. Previous studies by our research group revealed that premature leaf senescence in red raspberry significantly impairs fruit development, leading to smaller fruits and fewer drupelets per fruit, thereby reducing overall fruit quality [31]. Further investigations showed that most red raspberry cultivars exhibit premature aging during production, with ‘Polka’ displaying more pronounced symptoms than other varieties. Therefore, this dominant cultivar was selected as the focus of our study. Using integrated transcriptomic and metabolomic analyses, we identified key regulatory genes and metabolites associated with its leaf senescence. These findings offer technical support for developing preventive strategies to mitigate yield and quality losses caused by premature leaf aging in ‘Polka’.

## 2. Materials and Methods

### 2.1. Test Materials

The experimental materials were tissue-cultured seedlings of double-season red raspberry ‘Polka’, provided by Hebei Zishui Technology Co., Ltd., Xingtai, China. After hardening, they were transplanted into pots. When the seedlings reached ~50 cm in height, samples were collected for testing.

### 2.2. Experimental Design

**Figure 1:**
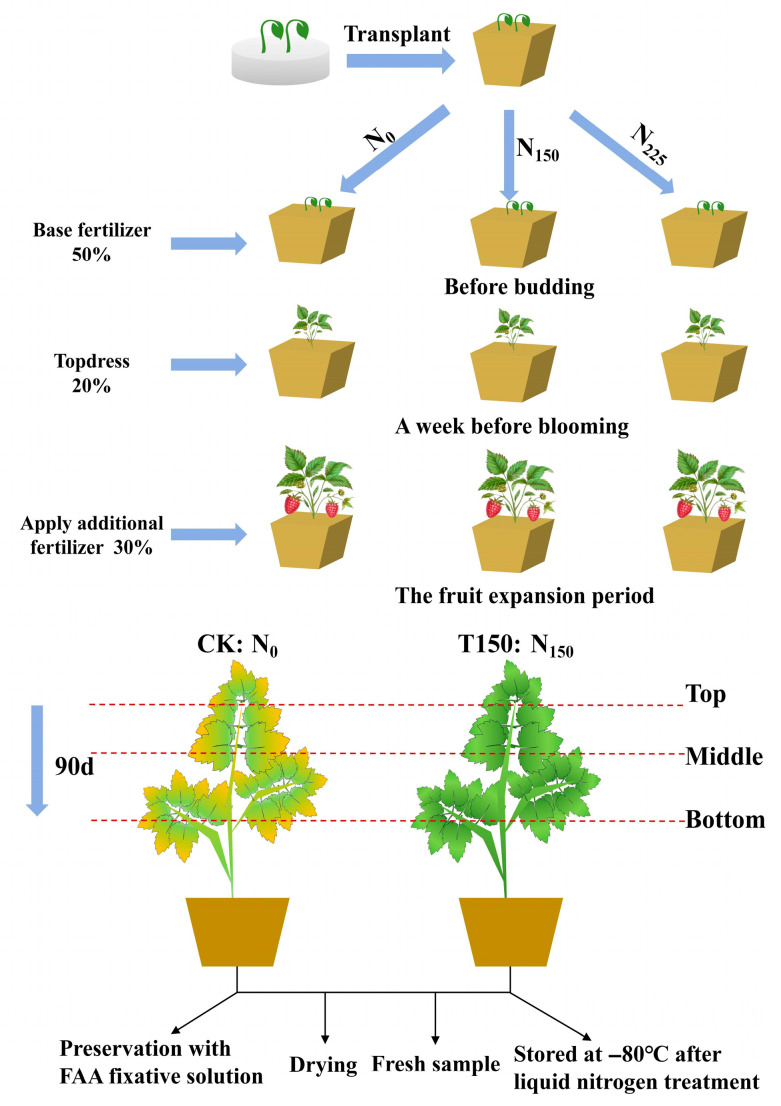
Experimental design process.

The experiment was conducted at the nursery of the Forestry College, Hebei Agricultural University, West Campus, Baoding City, Hebei Province (115°45′40″ E, 39°23′03″ N). The soil background nitrogen content was 1.19 ± 0.04 g/kg. The site has an average annual precipitation of 532 mm, a frost-free period of 180 days, and a continental monsoon climate. A controlled pot experiment was carried out. When the seedlings reached 15 cm in height, they were transplanted into 35 cm × 35 cm pots. Each pot was filled with 4 kg of dry loam soil sieved through a 2 mm mesh, and two seedlings were planted per pot. Three nitrogen treatments were applied, with 15 pots per treatment: 0 g, 0.10 g, and 0.15 g N·kg^−1^ dry soil, equivalent to field application rates of 0 kg·hm^−2^, 150 kg·hm^−2^, and 225 kg·hm^−2^, denoted as N_0_, N_150_, and N_225_, respectively. Nitrogen fertilizer was applied in three stages: 50% as basal fertilizer before germination, 20% one week before flowering, and 30% during the fruit expansion period. Based on prior results, N_0_ (no nitrogen) served as the control check (CK), while N_150_ (optimal nitrogen level) was designated as T150. All other management practices were consistent across treatments. Each treatment comprised 15 pots, divided into plots of 5 pots, with 3 replicates. Sampling was conducted 90 days after transplanting (Figure 1).

### 2.3. Determination Indicators and Methods

#### 2.3.1. Determination of Differences in Tree Growth and Physiological and Biochemical Indicators

##### Measurement of Tree Morphological Indicators

To determine the growth indicators of the two red raspberry seedling phenotypes, three biological replicates were set for each phenotype to ensure sample representativeness. The specific measurement methods were as follows:

Plant height, root length, and internode length were measured with a steel ruler (accuracy: 0.1 cm). The ground diameter was measured at the soil surface junction using a vernier caliper (accuracy: 0.01 mm). Root buds were counted manually by enumerating all visible buds on the roots of each seedling. Fresh weights were weighed using an electronic balance (accuracy: 0.001 g). Leaf thickness was measured using a YH-1 leaf thickness meter (Zhejiang Top Cloud-Agri Technology Co., Ltd., Hangzhou, China). The leaf SPAD value was measured using a YLS-4T chlorophyll detector (Sichuan Chengdu Yunzhice Technology Co., Ltd., Chengdu, China). Root activity was measured using the 2,3,5-triphenyl tetrazolium chloride (TTC) method [32].

##### Determination of Physiological and Biochemical Indicators

Ninety days after treatment, plants with uniform growth vigor were selected for leaf sampling for subsequent analyses. For each treatment, three red raspberry plants with basically consistent growth were chosen as biological replicates. From each plant, leaves from the upper, middle, and lower parts were sampled separately (i.e., samples from each leaf position from each biological replicate), and these samples were used for the determination of all physiological indices. Each leaf position sample from each biological replicate was subjected to three technical replicates to ensure the reliability of the results. After sampling, all physiological and biochemical indices were determined using a UV-1800 PC-type UV-visible spectrophotometer (Aoyi Instruments Co., Ltd., Shanghai, China). The specific methods for each index were as follows:

Chlorophyll and carotenoid contents: Determined via the acetone–ethanol mixture method (1:1 *v*/*v*). Fresh sampled leaves were rinsed with distilled water to remove surface impurities and blotted dry with filter paper, and 0.2 g of leaf tissue was weighed from each of the three biological replicates at the upper, middle, and lower leaf positions for each treatment. The leaf pieces were cut into small pieces, transferred to a centrifuge tube, and immersed in 20 mL of the acetone–ethanol mixture. They were then placed in a dark environment at 4 °C for 48 h for extraction until the leaf pieces turned white. Absorbance was measured at 665 nm, 649 nm, and 470 nm, with three technical replicates per sample (mean absorbance used for calculations), and contents were calculated using corresponding standard formulas based on absorbance values [32].

Soluble protein content: Determined using Coomassie Brilliant Blue G-250 staining. A total of 0.2 g of red raspberry leaf tissue was weighed, and a small amount of quartz sand and distilled water were added for grinding (to fully disrupt the tissue) to form a homogenate. The homogenate was processed following the Coomassie Brilliant Blue G-250 staining protocol, and absorbance was measured at 595 nm. Soluble sugar and starch contents: Measured using the anthrone colorimetric method. A total of 0.5 g of crushed dry red raspberry leaf tissue was weighed and subjected to extraction and treatment following this method, and absorbance was measured at 620 nm [33].

MDA content: Determined using the thiobarbituric acid (TBA) method. A total of 0.5 g of fresh red raspberry leaf tissue was weighed and treated by the TBA method, with absorbance measured at 450 nm, 532 nm, and 600 nm for content calculation [33].

SOD activity, POD activity, CAT activity, and superoxide anion content: For each index, 0.5 g of fresh leaf tissue was used, collected from the upper, middle, and lower parts (consistent with the sampling positions above), with three biological replicates per part. Prior to subsequent analyses, the samples were ground under low temperature conditions to form a homogenate to prevent enzyme inactivation. The homogenate was then used for subsequent determinations, with the specific methods as follows: SOD activity: determined via the nitroblue tetrazolium (NBT) photoreduction method, with absorbance measured at 560 nm [34]; POD activity: assayed using the guaiacol method; the increase in absorbance at 470 nm was recorded within 3 min, and activity was expressed as the change in absorbance per gram of fresh weight per minute [33]; CAT activity: measured via the hydrogen peroxide method, based on the decrease in absorbance of hydrogen peroxide at 240 nm [34]; superoxide anion content: determined using the hydroxylamine oxidation method, with absorbance measured at 530 nm for quantification [34].

#### 2.3.2. Slice Production Method

The leaves were cut into small pieces and fixed in formalin–aceto-alcohol (FAA) fixative [(50% ethanol, 90 mL) + glacial acetic acid (5 mL) + formaldehyde (5 mL)]. Plant tissues were embedded in paraffin, sectioned, and observed by double staining with safranin-fast green [35].

#### 2.3.3. Transcriptome Determination

##### RNA Extraction and Sequencing

To ensure experimental accuracy, the same batch of plant material used for physiological and biochemical measurements was selected. Three biological replicates were prepared and sent to Shanghai Paisonno Biotechnology Co., Ltd., Shanghai, China. RNA was extracted using the Tiangen RNAprep Pure Polysaccharide and Polyphenol Plant Total RNA Extraction Kit. A 300 bp fragment was selected, and RNA served as a template for cDNA synthesis. The first-strand cDNA was synthesized using 6-base random primers and reverse transcriptase; the second strand was synthesized using the first strand as a template. Following library construction, PCR amplification was used to enrich the library fragments. The library size was assessed using an Agilent 2100 Bioanalyzer (Agilent Technologies, Santa Clara, CA, USA), and total concentration was measured via fluorescence quantification. The high-quality reads, obtained by filtering raw data, were de novo assembled into transcripts. The longest transcript for each gene was designated as the Unigene for subsequent database annotation, differential expression analysis, and enrichment analysis. Genes with |log_2_(fold change)| ≥ 1 and p-adjust < 0.05 were considered significantly differentially expressed [36].

##### Real-Time Quantitative PCR Validation

To verify the analysis results, 12 differentially expressed genes were selected, and their expression levels were analyzed using qRT-PCR. A fluorescent dye was added to the PCR reaction system to monitor the fluorescence signal of each amplification cycle in real time, generating a fluorescence amplification curve used to quantify the initial template. Gene expression levels were calculated using the 2^−△△Ct^ method [37]. All samples were run in three technical replicates. The primers used are listed in Table S8.

#### 2.3.4. Metabolome Determination

##### Sample Extraction Method

To ensure experimental accuracy, test materials from the same batch used for physiological and biochemical measurements were selected, with six biological replicates per batch. The samples were sent to Shanghai Paisono Biotechnology Co., Ltd. (Shanghai, China) for extraction and analysis. After gradual thawing at 4 °C, an appropriate amount of sample was mixed with pre-cooled methanol/acetonitrile/water (2:2:1, *v*/*v*), vortexed, sonicated at low temperature for 30 min, left at −20 °C for 10 min, and centrifuged at 14,000× *g* at 4 °C for 20 min, and the supernatant was vacuum dried. For mass spectrometry analysis, 100 μL of aqueous acetonitrile was added to the dried extract and the mixture was centrifuged, and then the supernatant was used for analysis. Separation was performed using an Agilent 1290 Infinity LC ultra-high performance liquid chromatography (UHPLC) system with a C-18 column, and data were collected in both positive and negative ion modes using an AB Triple TOF 6600 mass spectrometer (AB SCIEX, Framingham, MA, USA) [38].

##### Data Analysis Process

The original data in Wiff format were converted to the mzXML format using ProteoWizard and processed with MS-DIAL software (a tool for untargeted metabolomics data analysis, Kanazawa University, Kanazawa, Japan, Version 4.80). This included peak alignment, retention time correction, and peak area extraction. The data extracted by MS-DIAL were first used for metabolite structure identification, followed by data preprocessing, experimental data quality evaluation, and finally data analysis. Data analysis included univariate and multivariate statistical analysis, differential metabolite screening, correlation analysis of differential metabolites, and Kyoto Encyclopedia of Genes and Genomes (KEGG) pathway analysis.

### 2.4. Data Processing and Analysis

**Figure 2:**
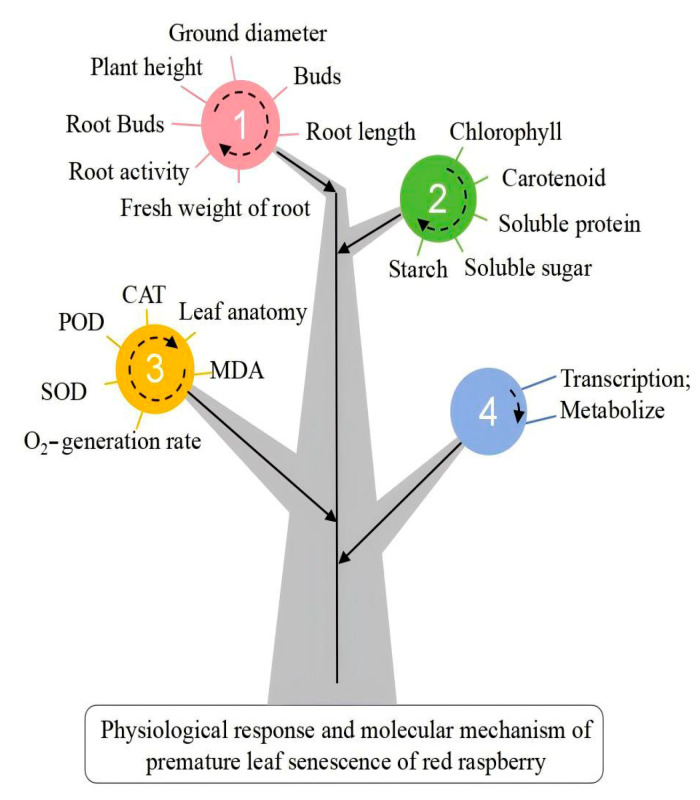
Relevant measurement indicators.

The measured index data (Figure 2) were analyzed using Microsoft Excel 2021 (Microsoft Corporation, Redmond, WA, USA) for membership function analysis and chart preparation. Significance analysis was conducted via one-way analysis of variance (ANOVA) using SPSS 26.0 (IBM Corporation, Armonk, NY, USA), and differences between T150 and CK were evaluated with the Tukey HSD multiple comparison method. Volcano and MA plots were generated using ggplot2 v3.2.1 (RStudio, Boston, MA, USA), and differential analysis was performed using DESeq 1.32 (Developed by EMBL, Heidelberg, Germany; and EBI, Cambridge, UK). Bar graphs were created using Origin 2024 (OriginLab Corporation, Northampton, MA, USA).

## 3. Results and Analysis

### 3.1. Growth of Red Raspberries at Three Nitrogen Application Levels

**Figure 3:**
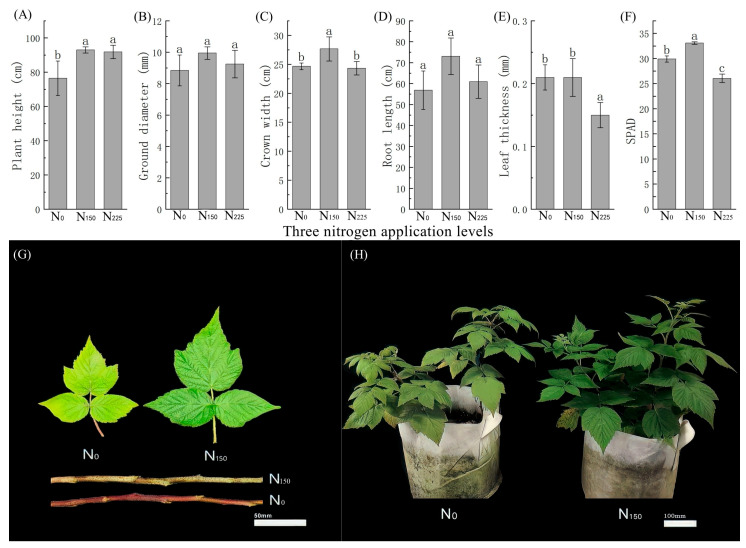
Phenotypic differences between premature senescent and normal red raspberry seedlings. (**A**) Plant height; (**B**) ground diameter; (**C**) crown width; (**D**) root length; (**E**) leaf thickness; (**F**) SPAD; (**G**) the lower leaves and branches of N0 and N150; (**H**) premature senescence red raspberry (**left**) and normal red raspberry (**right**). Note: (**G**) scale bar = 50 mm; (**H**) scale bar = 100 mm. Note: Different lowercase letters (a, b, c) indicate significant differences at *p* < 0.05 as determined by one-way ANOVA.

The plant exhibiting the weakest growth—characterized by short height, sparse crown, yellow leaves, and red petiole and stem—was defined as the early senescence phenotype plant (CK), while the plant with the best growth was defined as the normal-type red raspberry (T150). A study on the effects of three nitrogen application levels on red raspberry growth showed (Figure 3, Table S1) that N_150_ resulted in the highest plant height, ground diameter, crown size, root length, and SPAD (relative content of chlorophyll) value. The leaf thickness under N_150_ was similar to that of N_0_ and greater than that of N_225_. Under N_0_, yellowing and early senescence of lower leaves occurred at the budding stage, and the plant height, ground diameter, and root length were the lowest. Based on the membership function method, the growth ranking under the three nitrogen levels was N_150_ > N_0_ > N_225_. The plant with the best growth was designated as the normal-type red raspberry (T150), while N_0_, which showed premature leaf senescence and weak growth, was defined as the early senescence type red raspberry (CK).

### 3.2. Differences in Phenotypes and Physiological and Biochemical Parameters Between T150 and CK Plants

#### 3.2.1. Phenotypic Growth Differences Between T150 and CK

**Figure 4:**
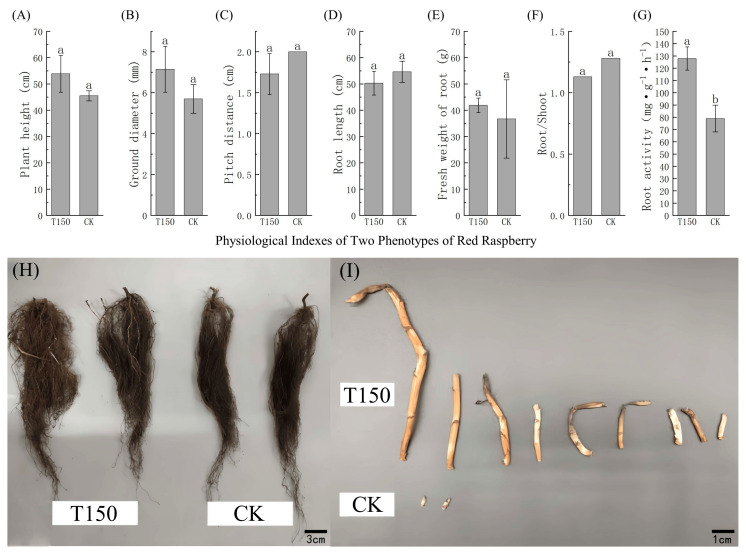
Growth traits and root morphology of red raspberry seedlings with normal and premature senescent leaves. (**A**) Plant height; (**B**) ground diameter; (**C**) pitch distance; (**D**) root length; (**E**) fresh weight of root; (**F**) root/shoot; (**G**) root activity; (**H**) root system differences between red raspberries with normal leaves and those with premature senescent leaves; (**I**) root buds of red raspberries with premature senescent leaves and those with normal leaves. Note: (**H**) scale bar = 3 cm; (**I**) scale bar = 1 cm. Note: Different lowercase letters (a, b) indicate significant differences at *p* < 0.05 as determined by one-way ANOVA.

Aboveground growth indices of T150 and CK plants were compared, and results showed that plant height, ground diameter, and number of buds were lower in CK than in T150. The external root morphology of T150 and CK plants was also analyzed (Figure 4, Table S2), revealing that the root length of CK was 4 cm longer than that of T150, but its fresh weight was 5.2 g lower. The root activity in CK was significantly lower than in T150, and the number of root buds in T150 was significantly higher than in CK.

#### 3.2.2. Differences in Photosynthesis and Osmotic Regulation Substances in Leaves of T150 and CK Plants

**Figure 5:**
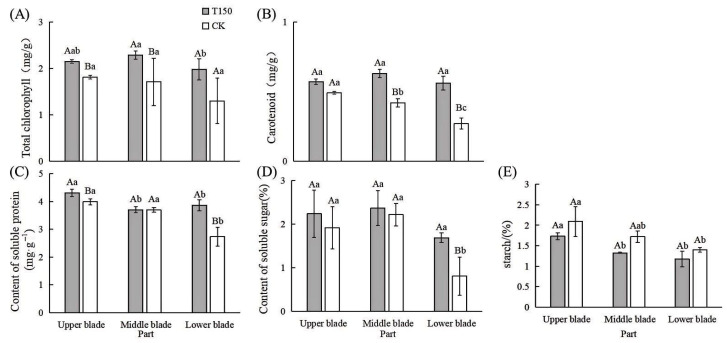
Contents of photosynthetic pigments and osmoregulatory substances in different parts of leaves of normal and premature senile raspberry. (**A**) Total chlorophyll; (**B**) carotenoid; (**C**) soluble protein content; (**D**) soluble sugar content; (**E**) starch content. Note: Capital letters represent the difference between normal type and premature senescence type, and lowercase letters represent the difference of all parts, which is significant at the level of *p* < 0.05. The same as below.

The total chlorophyll, carotenoids, chlorophyll a, and chlorophyll b contents in leaves from different parts of T150 and CK plants are shown in Table S3. The most pronounced differences between T150 and CK were observed in the middle and lower leaf sections. In the lower leaves, the total chlorophyll content in T150 was 0.68 mg/g higher than that in the lower leaves of the premature senescence phenotype (Figure 5A,B, Table S3), and carotenoid content was 0.29 mg/g higher than in the corresponding CK leaves. Among both phenotypes, the chlorophyll content was lowest in the lower leaves. The contents of carotenoid and chlorophyll a also showed significant differences across different leaf positions in both phenotypes, with distribution trends similar to that of total chlorophyll.

Leaves from different parts of T150 and CK plants were also analyzed for soluble protein content. The results (Figure 5C) show that soluble protein levels were highest in the upper young leaves for both phenotypes—4.31 mg/g in T150 and 3.99 mg/g in CK. In CK, the lower leaves contained 2.73 mg/g of soluble protein, significantly lower than in other parts. No significant difference was observed in the soluble protein content of the middle leaves between the two phenotypes.

The soluble sugar contents in the upper, middle, and lower leaves of T150 were 0.32%, 0.15%, and 0.88% higher, respectively, than those in the corresponding parts of CK (Figure 5D).

The starch content was highest in the upper leaves and lowest in the lower leaves of both phenotypes (Figure 5E). In T150, starch levels in the upper, middle, and lower leaves were 0.36%, 0.40%, and 0.23% lower, respectively, than those in the same parts of CK.

#### 3.2.3. Changes in Membrane Lipid Peroxidation and Antioxidant Enzyme Activities in Leaves of T150 and CK Plants

**Figure 6:**
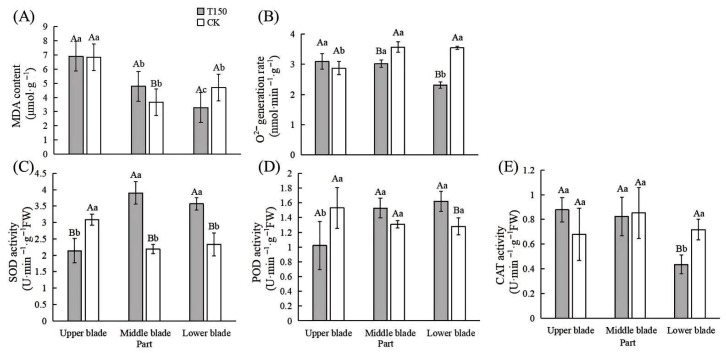
Changes in membrane lipid peroxidation and antioxidant enzyme activities in two phenotypes of red raspberry. (**A**) MDA content; (**B**) O^2−^ generation rate; (**C**) SOD activity; (**D**) POD activity; (**E**) CAT activity. Note: Capital letters represent the difference between normal type and premature senescence type, and lowercase letters represent the difference of all parts, which is significant at the level of *p* < 0.05.

The MDA content of leaves from different parts of T150 and CK is shown in Figure 6A. In T150, MDA content gradually decreased from the top to the bottom. In CK, the MDA content was lowest in the middle leaves and highest in the upper leaves. Comparing the two red raspberry phenotypes, the MDA content in the upper leaves of T150 was not significantly different from that of CK. However, the middle leaves of T150 showed an MDA content that was 1.13 μmol·g^−1^ higher than CK, while the lower leaves were 1.41 μmol·g^−1^ lower. The superoxide anion generation rate in different parts of T150 and CK is shown in Figure 6B. In T150, the superoxide anion generation rate decreased progressively from the upper to the lower parts. In CK, the generation rate was highest in the middle leaves, not significantly different from the lower leaves, and lowest in the upper leaves. Compared with CK, the upper leaves of T150 showed a significantly higher superoxide anion generation rate, while the middle and lower leaves had significantly lower rates, by 0.55 and 1.23 nmol·min^−1^·g^−1^, respectively.

Leaves from various parts of T150 and CK were also analyzed for antioxidant enzyme activities. The results are shown in Figure 6C, Table S9. In T150, SOD activity was highest in the middle leaves at 3.90 U·min^−1^·g^−1^ FW, and lowest in the upper leaves. In CK, SOD activity was highest in the upper leaves at 3.09 U·min^−1^·g^−1^ FW, and lowest in the middle leaves. The SOD activity of the lower leaves in T150 was 1.24 U·min^−1^·g^−1^ FW, which was higher than that in CK. As shown in Figure 6D, POD activity in the middle and lower leaves of T150 was significantly higher than in CK, while in the upper leaves, it was significantly lower. The CAT activity in T150 leaves gradually decreased from the upper to the lower part (Figure 6E). The CAT activity in the middle leaves of T150 was slightly higher than in CK by 0.03 U·min^−1^·g^−1^ FW, while in the lower leaves, it was 0.28 U·min^−1^·g^−1^ FW lower than in CK.

#### 3.2.4. Differences in Leaf Anatomical Structure Between T150 and CK Plants

**Figure 7:**
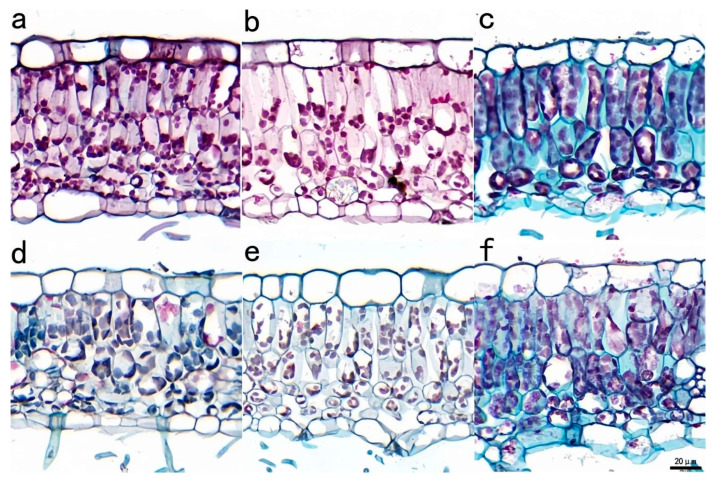
Anatomical structure of normal and premature senescence raspberry leaves. Note: (**a**): normal upper part; (**b**): normal middle part; (**c**): normal lower part; (**d**): premature senescence upper part; (**e**): premature senescence middle part; (**f**): premature senescence lower part; scale bar = 20 µm.

The anatomical structure of T150 and CK leaves is shown in Figure 7. The epidermis comprised a single layer of flat, tightly arranged thin-walled cells covering both the ventral and dorsal leaf surfaces. The outer wall of the upper epidermis was thicker, while that of the lower epidermis was thinner. In the palisade tissue of the upper and middle leaves of T150, chloroplasts were positioned close to the cell wall, whereas in CK, they were more diffusely arranged. The spongy tissue cells were irregular in shape, and in the lower leaves of CK, they were loosely arranged with large intercellular spaces.

### 3.3. Transcriptome Data Analysis of Lower Leaves of T150 and CK Plants

#### 3.3.1. Transcriptome Sequencing Data Results and Unigene Functional Annotation and Analysis

The differences in physiological indicators between T150 and CK showed that the chlorophyll, carotenoids, soluble protein, sugar content, and SOD and POD activities in the lower leaves of T150 were significantly higher than those in CK red raspberries. In contrast, the starch, MDA content, superoxide anion generation rate, and CAT activity in CK leaves were significantly higher than those in T150. Based on these differences, the lower leaves of T150 and CK were selected for joint transcriptome and metabolome analysis.

Cutadapt software (Version 4.4, University of Lausanne, Lausanne, Switzerland) was used to remove reads containing 3′ adapter sequences and those with an average quality score < Q20. As shown in Figure S1A and Table S4, the percentage of Clean Reads across the six samples ranged from 90.16% to 91%. Q20 values were above 97%, and Q30 values exceeded 94%, indicating high sequencing accuracy. Gene function annotation was performed using Unigene (Figure S1B), with reference to several databases, including NR, GO, KEGG, eggNOG, Swiss-Prot, and Pfam. The highest number of annotated unigenes was found in the NR database (25,123; 67.35%), followed by eggNOG (22,975; 61.59%), Swiss-Prot (20,379; 54.63%), Pfam (16,023; 42.95%), and GO (14,786; 39.64%). The fewest annotations were in KEGG, with 11,014 unigenes (29.52%). Only 5843 unigenes (15.66%) were annotated across all databases. Annotation using the NR database also enabled comparison of gene sequence similarity with closely related species, providing functional insights into the genes of this species. As shown in Figure S1C, 54.66% of unigene sequences matched *Rosa chinensis*, followed by matches with *Fragaria vesca* subsp. *vesca*, *Carpinus fangiana*, and other species.

#### 3.3.2. Analysis of Differentially Expressed Genes

**Figure 8:**
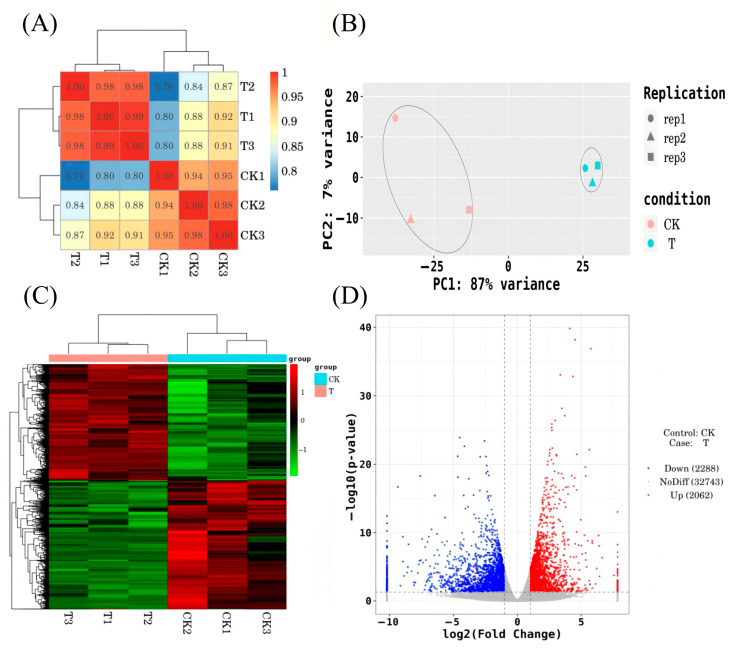
Correlation analysis and characterization of DEGs. (**A**) Sample correlation test; (**B**) PCA principal components; (**C**) differentially expressed gene clustering; (**D**) volcano plot of differentially expressed genes. Note: T = T150. (**C**) Red indicates high-expression genes and green indicates low-expression genes; (**D**) red dots indicate upregulated genes in this group, blue dots indicate downregulated genes in this group, and grey dots indicate non-significant differentially expressed genes.

The Pearson correlation coefficient was used to assess gene expression similarity between samples; values closer to 1 indicate higher similarity (Figure 8A). Principal component analysis (PCA) was used to cluster similar samples, with shorter distances indicating greater similarity (Figure 8B).

Clustering was performed based on the expression levels of the same gene across different samples and the expression patterns of different genes within the same sample. Euclidean distance was used for calculating similarity, and hierarchical clustering was conducted using the complete linkage (longest distance) method. The results are shown in Figure 8C. Differentially expressed genes (DEGs) were identified using the criteria |log_2_FoldChange| > 1 and *p*-value < 0.05. A volcano plot of DEGs was generated using the ggplot2 package in R. In the plot, the two vertical dotted lines represent the twofold expression difference threshold, while the horizontal dotted line marks the *p*-value = 0.05 threshold. The blue dots indicate downregulated genes compared to CK, red dots represent upregulated genes, and gray dots denote genes without significant differential expression. The results are shown in Figure 8D, where a total of 4350 DEGs were identified, including 2062 upregulated and 2288 downregulated genes, accounting for 47.4% and 52.6% of the total, respectively.

#### 3.3.3. KEGG and GO Enrichment Analysis of Differentially Expressed Genes

**Figure 9:**
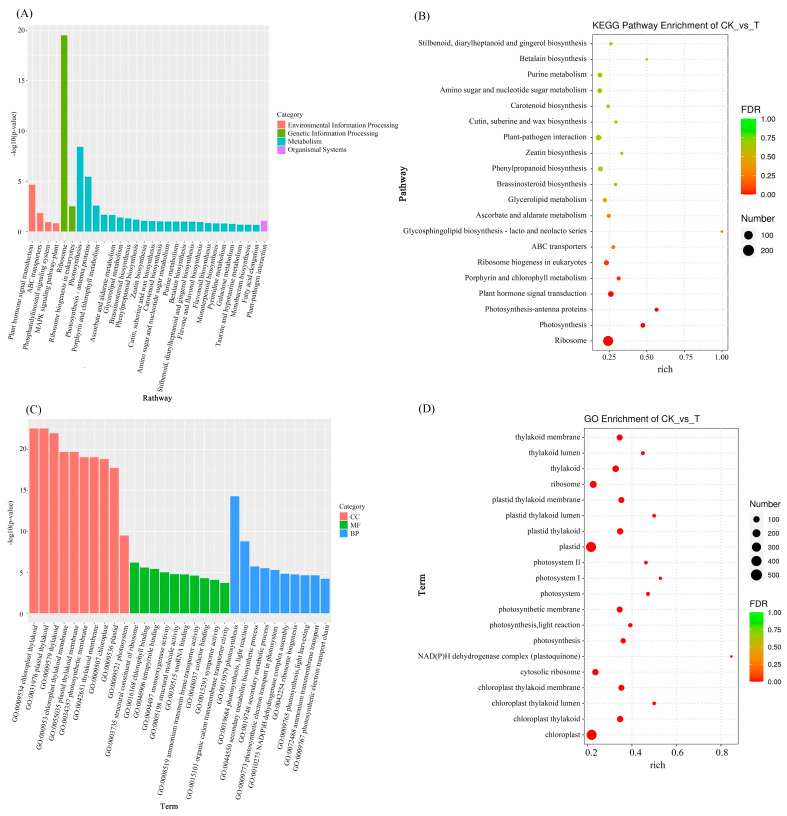
KEGG and GO enrichment analysis of DEGs between two red raspberry phenotypes. (**A**) KEGG pathway enrichment histogram; (**B**) KEGG pathway enrichment bubble plot of DEGs between the two phenotypes; (**C**) GO term enrichment histogram; (**D**) GO term enrichment scatter plot of DEGs between the two phenotypes. Note: T = T150.

To clarify the main pathways involved in leaf senescence in red raspberry seedlings, the DEGs were mapped to the KEGG database (Figure 9A and Table S10). An enrichment scatter plot of the top 20 KEGG pathways was generated (Figure 9B). The annotation results indicated that the most enriched pathway was ribosome, followed by photosynthesis, photosynthesis-antenna proteins, plant hormone signal transduction, and porphyrin and chlorophyll metabolism. In the ribosome pathway, the number of DEGs was highest, with 281 genes—49 upregulated and 232 downregulated.

To explore the main biological functions of DEGs, topGO was used to perform GO enrichment analysis on the DEGs in normal and prematurely aged leaves of red raspberry seedlings (significant enrichment was defined as *p* < 0.05). As shown in Figure 9C, these genes were categorized into three groups based on GO analysis: cellular component (CC), molecular function (MF), and biological process (BP). In terms of cellular components, chloroplast thylakoid, plastid thylakoid, and its thylakoid were significantly enriched. For molecular function, the DEGs were mainly associated with structural constituent of ribosome, followed by chlorophyll binding and tetrapyrrole binding. Within biological processes, the genes were primarily enriched in photosynthesis, light reaction, and the secondary metabolite biosynthetic process (Figure 9D).

#### 3.3.4. Physiological and Biochemical Indicators and Hormone-Related Differentially Expressed Genes

**Figure 10:**
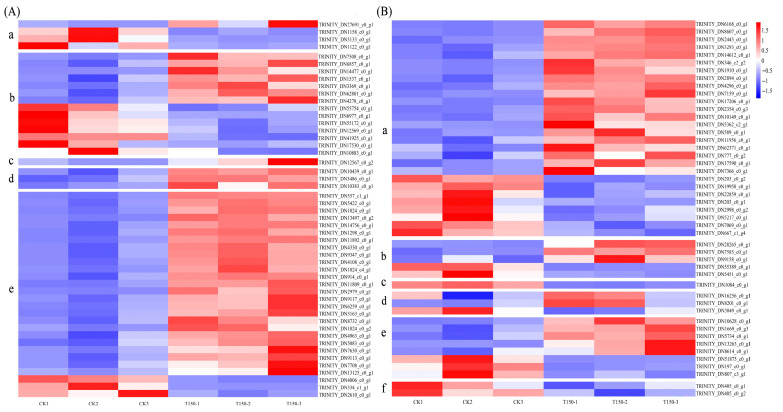
Heatmap of physiological- and hormone-related DEGs between two red raspberry phenotypes. (**A**) Differential expression genes related to physiological indicators of two phenotypes of red raspberries. a: senescence-related. b: POD-related. c: CAT-related. d: starch-related. e: chlorophyll-related; (**B**) hormone related differentially expressed genes in two phenotypes of red raspberries. a: auxin-related. b: cytokinins-related. c: gibberellin-related. d: ABA-related. e: ethylene-related. f: brassinolide-related; Note: Red indicates upregulated genes, and blue indicates downregulated genes.

DEGs related to physiological indicators of early senescence in red raspberry leaves were identified, and the results are shown in Figure 10A. Four DEGs were related to senescence—one was upregulated and three were downregulated. Fourteen DEGs were related to peroxidase activity, with seven upregulated and seven downregulated. One catalase-related gene was identified and was upregulated. Three starch-related genes were detected, all of which were upregulated. Twenty-eight chlorophyll-related genes were identified, of which 25 were upregulated and 3 were downregulated.

DEGs associated with hormone regulation were also screened, and the results are presented in Figure 10B. Twenty-eight DEGs were related to auxin, with 20 upregulated and 8 downregulated. Five DEGs were related to cytokinin, including three upregulated and two downregulated. One gibberellin-related gene was found to be downregulated. Three genes were associated with abscisic acid, with two upregulated and one downregulated. Eight DEGs were related to ethylene, including five upregulated and three downregulated. Two brassinolide-related DEGs were identified, both of which were downregulated.

To verify the results of DEGs involved in red raspberry leaf senescence, 12 were selected for quantitative fluorescence PCR validation, including 6 upregulated and 6 downregulated genes. As shown in Figure S1E and Table S11E, the ordinate represents the RNA-seq expression levels, and the abscissa represents the qRT-PCR expression levels. The results showed that the expression trends of these 12 DEGs were consistent with the transcriptome sequencing data, confirming that transcriptome sequencing is efficient and accurate. This approach can therefore be used to screen DEGs and analyze red raspberry leaf senescence, enhancing the reliability of the experiment.

### 3.4. Metabolome Data Analysis of Lower Leaves of T150 and CK Plants

#### 3.4.1. Metabolite PCA and OPLS-DA Analysis

A total of 1292 metabolites were identified in this experiment, including 793 in positive ion mode and 499 in negative ion mode. The metabolites were classified and quantified based on their chemical categories, and the proportion of each type is shown in Figure S1D.

To understand the overall distribution trends of the two red raspberry phenotypes, PCA was conducted. The two treatments formed two distinct clusters (Figure S2A,B), effectively differentiating the groups. This result indicates good experimental repeatability and significant differences between the two sample sets.

The data from T150 and CK treatments were further analyzed using orthogonal partial least squares discriminant analysis (OPLS-DA), which filters out irrelevant variation and accurately highlights intergroup differences. The resulting score plots (Figure S2C,D) illustrate both inter- and intra-group variation and clearly separate the two groups. Evaluation parameters R^2^ X, R^2^ Y, and Q^2^ were obtained via cross-validation. The closer these values are to 1, the more stable and reliable the model. Generally, Q^2^ > 0.5 indicate an effective model, while Q^2^ < 0.3 suggest low reliability. In this analysis, Q^2^ values were 0.993 in positive ion mode and 0.996 in negative ion mode, indicating highly robust models.

A permutation test was performed to validate the model. In the plot, the horizontal axis represents permutation order, the vertical axis shows R^2^ (green dots) and Q^2^ (blue dots), and the dotted line represents the regression line for both parameters. As shown in Figure S2E,F, R^2^ and Q^2^ values of the randomized models decreased as permutation order decreased, confirming the strong robustness of the original model.

#### 3.4.2. Screening of Differentially Expressed Metabolites (DEMs)

Metabolites associated with premature senescence in red raspberry leaves were screened using OPLS-DA, with variable importance in projection (VIP) > 1 and *p*-value < 0.05 as selection criteria. A total of 135 significantly differential metabolites were identified (87 in positive ion mode and 48 in negative ion mode), including 51 lipids and lipid-like molecules; 27 phenylpropanoids and polyketides; 13 organic oxygen compounds; 9 organic heterocyclic compounds; 7 organic acids and their derivatives; 5 benzene ring compounds; 2 organic nitrogen compounds; 2 alkaloids and their derivatives; 2 lignin, neolignin, and related compounds; and 17 other substances. Among these, 60 metabolites (19 negative and 41 positive ions) were significantly upregulated in T150, accounting for 44.44%, while 75 metabolites (29 negative and 46 positive ions) were significantly downregulated, accounting for 55.56%. Tables S5 and S6 list the top 20 differential metabolites based on fold change in expression for positive and negative ion modes, respectively.

**Figure 11:**
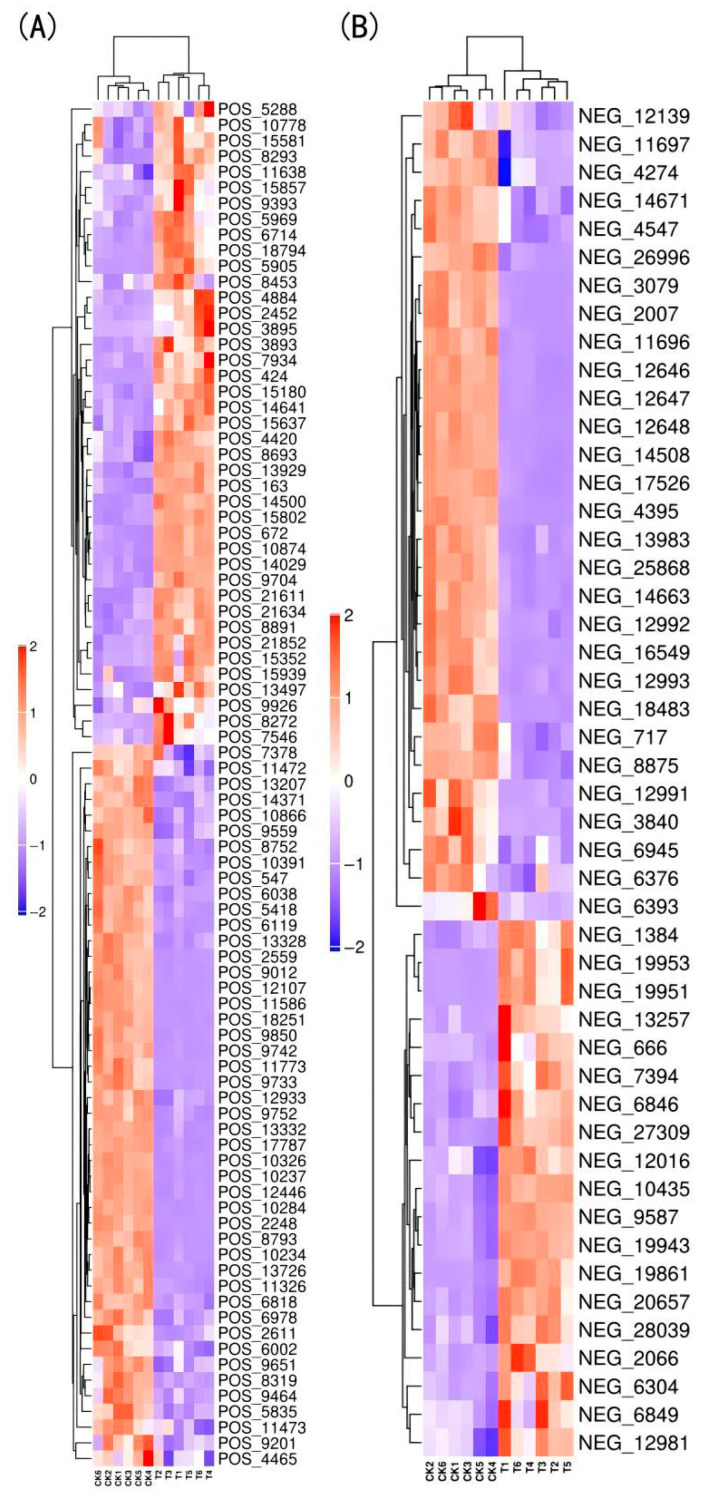
Hierarchical clustering heatmap of significantly different metabolites between two red raspberry phenotypes. (**A**) Positive ion mode; (**B**) negative ion mode. Note: red indicates upregulated metabolites, blue indicates downregulated metabolites; T = T150.

The samples and differential metabolites were clustered, with red indicating significantly upregulated metabolites and blue indicating significantly downregulated metabolites. As shown in Figure 11, the two phenotypes of red raspberry samples clustered separately, indicating distinct metabolic profiles between the two groups.

#### 3.4.3. KEGG Pathway Analysis

**Figure 12:**
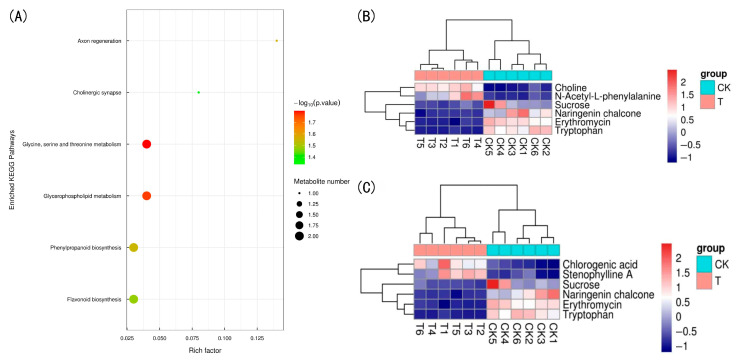
KEGG enrichment pathway diagram and differential metabolite cluster heat map. (**A**) KEGG enrichment pathway diagram of differential metabolites of two phenotypic red raspberries; (**B**) biosynthesis of secondary metabolites; (**C**) metabolic pathways. Note: T = T150.

To further investigate metabolic differences, pathway enrichment analysis was performed using the KEGG database on the differential metabolites of the two phenotypes. As shown in Figure 12A, six pathways with *p*-value < 0.05 were identified: Glycine, serine and threonine metabolism; glycerophospholipid metabolism; phenylpropanoid biosynthesis; flavonoid biosynthesis; axon regeneration; and cholinergic synapse.

The differential metabolites from both positive and negative ion modes were merged and annotated using the KEGG database (VIP > 1, *p*-value < 0.05). The identified metabolites were mapped to 34 pathways, with the largest number annotated to secondary metabolite biosynthesis and general metabolic pathways (six metabolites each). These were visualized using a heatmap, where each row represents a differential metabolite and each column represents a sample. As shown in Figure 12B,C, CK and T150 samples clustered together. Compared with CK, chlorogenic acid and choline were significantly upregulated in T150, while naringenin chalcone, erythromycin, and tryptophan were significantly downregulated.

### 3.5. Joint Analysis of Metabolome and Transcriptome of Lower Leaves of T150 and CK Plants

For the joint analysis of the metabolome and transcriptome, we first obtained quantitative detection and analysis results for both datasets and mapped the transcripts corresponding to differential metabolites and related enzymes to the KEGG database. Transcriptomic and metabolomic data were cross-validated to identify metabolic pathways with significant changes. The results (Table S7) showed that the differential metabolites and transcripts were annotated to the phenylpropanoid biosynthesis (ko00940) and flavonoid biosynthesis (ko00941) pathways. In the phenylpropanoid biosynthesis pathway, 31 genes were differentially expressed, including 10 upregulated and 21 downregulated genes. Two differential metabolites—chlorogenic acid and stenophylline A—were identified. In the flavonoid biosynthesis pathway, eight genes were differentially expressed, with five upregulated and three downregulated. Two differential metabolites—chlorogenic acid and naringenin chalcone—were identified.

**Figure 13:**
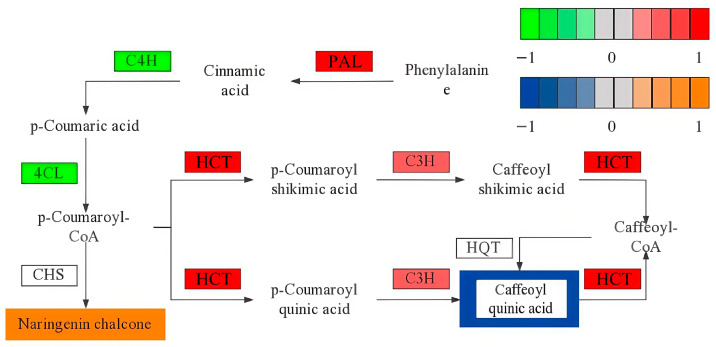
Key pathway of red raspberry premature senescence.

The metabolic pathway analysis results were visualized, with differential metabolite expression indicated by a color gradient from blue (low) to orange (high), and transcript expression by a gradient from green (low) to red (high). The phenylpropanoid biosynthesis pathway serves as a key upstream pathway of flavonoid biosynthesis. As shown in Figure 13, in CK leaves, the contents of phenylalanine ammonia lyase (PAL), shikimate O-hydroxycinnamoyltransferase (HCT), and 5-O-(4-coumaryl)-D-quinic acid 3′-monooxygenase (C3H) increased, while the contents of trans-cinnamate 4-monooxygenase (C4H) and 4-carboxymethyl-CoA ligase (4CL) decreased. The naringenin chalcone content increased, whereas that of chlorogenic acid decreased.

## 4. Discussion

### 4.1. Apparent Differences and Physiological and Biochemical Changes Between T150 and CK

Plants undergo dramatic changes in external morphology as well as internal physiological and biochemical processes during senescence. The green-type wheat maintains higher chlorophyll content, net photosynthetic rate, and antioxidant enzyme activity during the late stages of leaf senescence [39]. Low nitrogen levels induce senescence in maize seedlings, leading to reduced leaf area, chlorophyll content, and net photosynthetic rate, as well as decreased accumulation of soluble sugars and starch [40]. The results of this experiment are consistent with the findings reported above. Compared with normal leaves, prematurely senescent raspberry seedlings show significantly reduced plant height, ground diameter, and number of buds, indicating that the seedlings may lack sufficient nutrients to support aboveground growth. The chlorophyll content decreases progressively from the upper to the lower parts of the plant, and the lower leaves turn yellow, indicating an aging state. In some prematurely aging trees, both upper and lower leaves fall off, further indicating weakened photosynthetic capacity, which impairs the formation of photosynthetic products.

Appropriate nitrogen fertilizer supplementation can improve leaf function. High-temperature stress causes premature senescence in wheat flag leaves, reducing starch accumulation and the activity of key enzymes involved in starch synthesis. Nitrogen application can enhance photosynthetic efficiency in leaves, accelerate starch synthesis in grains, and thereby increase yield [41]. Plants use photosynthesis to synthesize starch, which is stored as a polysaccharide in fruits, seeds, and rhizomes. In this experiment, leaf starch content was lowest and decreased from the upper to the lower part of the plant. This was likely due to the reduced photosynthetic capacity of lower leaves, resulting in decreased starch synthesis. The starch content in CK leaves was higher than that in the corresponding parts of T150, while soluble sugar content was lower. This may have been due to reduced metabolic consumption in the plant, preventing timely starch degradation and utilization, thus causing accumulation. Alternatively, it may reflect the transport of soluble sugars to other organs, where they are converted into starch and stored.

Topdressing with nitrogen fertilizer can increase the activity of SOD and CAT in leaves, reduce membrane lipid peroxidation and malondialdehyde (MDA) accumulation, maintain flag leaf greenness for a longer period, and enhance production potential [42]. The SOD and POD activities in the middle and lower leaves of T150 were significantly higher than those in CK, while CAT activity in CK leaves was significantly higher than in T150. MDA and superoxide anion contents in the lower leaves of CK were significantly higher than those in T150, indicating that the antioxidant system in the middle and lower leaves of T150 removed harmful substances more efficiently than they were produced, thereby protecting cellular structure and function while delaying premature senescence. In conclusion, the premature senescence of red raspberries may be due to the excessive accumulation of MDA and O_2_^−^, reduced antioxidant enzyme activity, and impaired self-regulation resulting from malnutrition.

Studies have shown that an unbalanced root-to-shoot relationship can contribute to plant aging [43]. When stimulated, plant roots can adjust to environmental changes by increasing the root-to-shoot ratio [44], as well as improving root quality [45]. Low nitrogen levels induced senescence in maize seedlings, yet still led to increases in root dry weight, total root length, and average root diameter [40]. Under low nitrogen conditions, the elongation and maturation zones of rapeseed roots exhibit active cell division and elongation, while the root-to-shoot ratio and nitrogen use efficiency also increase significantly [46,47]. Wheat root activity is strongly correlated with leaf senescence index [48], indicating that root systems may influence leaf senescence. In this experiment, the roots of CK seedlings were significantly longer than those of T150, indicating that CK seedlings allocated more photosynthates to root growth, which promoted preferential root development and improved access to water and nitrogen in the soil, thereby enhancing nutrient use efficiency. However, the number of root buds in CK was lower than in T150, possibly because nutrients were primarily allocated for root growth and partially reserved for future bud germination. The root activity in the premature senescence phenotype (CK) was lower than in T150, suggesting that the CK root system had a weaker capacity to absorb soil water and nutrients, resulting in insufficient supply to the aboveground parts of the plant.

Leaf senescence is regulated internally, beginning at the base of the leaves and gradually progressing to the upper leaves [49]. In this experiment, the chloroplasts in the palisade tissue of the upper and middle leaves of T150 were located near the cell wall, whereas in CK, the chloroplasts were more diffusely distributed. This distribution pattern in T150 reflects its stronger regulatory capacity. In contrast, the chloroplasts in the lower leaves of both T150 and CK were dispersed throughout the cytoplasm, allowing more effective use of scattered light to enhance photosynthesis. The spongy tissue cells in the lower leaves of CK were loosely arranged, with large intercellular spaces that facilitated gas exchange with the external environment.

### 4.2. Analysis of Differentially Expressed Genes in Premature Leaf Senescence of Red Raspberry

Plant senescence involves physiological, biochemical, and molecular biological processes, regulated by multiple genes. In this study, lower leaves displaying premature senescence and normal phenotypes in raspberry were analyzed de novo, yielding 37,304 transcripts. The majority of unigenes were annotated in the NR database. Annotation results indicated functional similarity with roses and wild strawberries within the Rosaceae family. A total of 4350 DEGs were identified, including 2062 upregulated and 2288 downregulated. GO enrichment analysis showed that DEGs were associated with chloroplast thylakoids, plastid thylakoids, and thylakoids in the cellular component category; ribosomal structural components, chlorophyll binding, and tetrapyrrole binding in the molecular function category; and photosynthesis, light reactions, and secondary metabolite biosynthesis in the biological process category. These findings suggest that premature senescence in red raspberries primarily affects chloroplast regulation, as well as transcriptional and translational processes.

In this experiment, the senescence-related genes *TRINITY_DN1158_c0_g1*, *TRINITY_DN3133_c0_g1*, and *TRINITY_DN1122_c0_g1* were upregulated in prematurely senescent red raspberry. In contrast, in the normal phenotype, the upregulated expression of chlorophyll-binding protein genes *TRINITY_DN557_c1_g1*, *TRINITY_DN5422_c0_g1*, and *TRINITY_DN1024_c0_g1*; chlorophyll reductase genes *TRINITY_DN8732_c0_g1* and *TRINITY_DN7630_c0_g1*; the chlorophyll oxygenase gene *TRINITY_DN5083_c0_g1*; and the light-harvesting chlorophyll protein complex gene *TRINITY_DN6259_c0_g1* supported normal photosynthesis. Additionally, peroxidase-related genes *TRINITY_DN7508_c0_g1*, *TRINITY_DN14477_c0_g1*, *TRINITY_DN3369_c0_g1*, and others were upregulated, helping reduce oxidative damage to cells. In this experiment, the starch synthase genes *TRINITY_DN10439_c0_g1*, *TRINITY_DN3486_c0_g1*, and *TRINITY_DN10383_c0_g1* were upregulated in the normal phenotype, ensuring stable photosynthesis and starch synthesis.

### 4.3. Analysis of Differential Metabolites in Premature Senescence of Red Raspberry Leaves

Metabolomics was used to quantitatively analyze all metabolites in the plants, revealing their relationship to physiological changes. A total of 1292 metabolites were identified, including 793 positive ions and 499 negative ions. Among these, 135 significantly different metabolites (87 positive ions and 48 negative ions) were identified based on VIP > 1 and *p* < 0.05. These included 51 lipids and lipid molecules, 27 phenylpropanes and polyketides, 13 organic oxygen compounds, 9 organic heterocyclic compounds, 7 organic acids and derivatives, 5 benzene ring compounds, 2 organic nitrogen compounds, 2 alkaloids and derivatives, 2 lignin- and neolignin-related compounds, and 17 other substances. Clustering analysis of all samples and differential metabolites showed that the T150 group and CK group formed distinct clusters, indicating that the identified differential metabolites can effectively distinguish between the two groups. These metabolites were annotated to 34 pathways. Notably, the secondary metabolite biosynthesis (ko01110) and metabolic pathways (ko01100) each contained six annotated differential metabolites, suggesting their important roles in the premature senescence of red raspberry.

Secondary metabolites are small organic compounds synthesized via complex secondary metabolic pathways during plant growth and development. They exist in various forms but are generally low in concentration [50]. Terpenoids refer broadly to all isoprene polymers and their derivatives [51]. Among terpenes, monoterpenes and sesquiterpenes are the main components of volatile oils, diterpenes are the primary constituents of resins, and triterpenes commonly occur as saponins [52]. Saponins are the main active components of many Chinese herbal medicines (such as ginseng, licorice, bupleurum, etc.), known for their antibacterial, antipyretic, and anticancer properties. Studies have shown that triterpenoid compounds in raspberries possess antioxidant and antibacterial activities [53]. Phenols include both flavonoids and non-flavonoids. Flavonoids comprise flavones, dihydroflavonoids, chalcones, anthocyanidins, etc. [54]. Nitrogen-containing compounds mainly consist of alkaloids, non-protein amino acids, and cyanogenic glycosides, most of which serve defensive roles. In this experiment, metabolomic analysis identified 20 triterpenoid metabolites, 19 of which were upregulated in CK raspberries. Additionally, 19 flavonoids were detected, of which 15 were downregulated. These results suggest that different premature senescence phenotypes in red raspberries employ distinct strategies to mitigate environmental stress. CK may enhance its antibacterial and antiviral capacity by generating terpenoid metabolites, while T150 appears to bolster its antioxidant defenses via flavonoid synthesis, thereby alleviating premature senescence.

### 4.4. Analysis of Key Pathways in Premature Senescence of Red Raspberry Leaves

Phenylpropanoid biosynthesis is an important secondary metabolic pathway in plants [55]. Red pear enhances its antioxidant capacity through phenolic compounds produced in this pathway [56]. The phenylpropanoid biosynthesis pathway also serves as a key upstream pathway of flavonoid biosynthesis [57]. Studies have shown that overexpression of phenylalanine ammonia-lyase (*PAL*), cinnamate 4-hydroxylase (*C4H*), 4-coumarate:coenzyme A ligase (*4CL*), hydroxycinnamoyl transferase (*HCT*), hydroxycinnamoyl-CoA:quinate hydroxycinnamoyl transferase (*HQT*), and coumarate 3-hydroxylase (*C3H*)-related genes can significantly increase the chlorogenic acid content in cells [58].

Chlorogenic acid, a caffeoylquinic acid compound, is widely found in higher dicotyledonous plants and ferns [59]. Chlorogenic acid possesses antibacterial properties and is widely used in medicine to reduce inflammation [60]. One of chlorogenic acid’s most important biological activities is antioxidant activity [61]. In this experiment, senescent leaves had lower chlorogenic acid content compared to normal leaves, and thus had reduced capacity to eliminate O_2_^−^, ultimately leading to cell damage and subsequent leaf senescence.

The flavonoid biosynthesis pathway is ubiquitous in plants and produces various secondary metabolites, known as flavonoid compounds [62], which contribute significantly to combating chronic diseases. This pathway also plays an important role in plant growth and development. In plants, flavonoids have antioxidant functions; scavenging ROS, they enhance stress resilience and influence growth and development through the synthesis and distribution of auxins [63]. In Chinese cabbage, melatonin treatment significantly delays leaf senescence, preserving high quality and maintaining elevated total phenolic and flavonoid contents [64].

**Figure 14:**
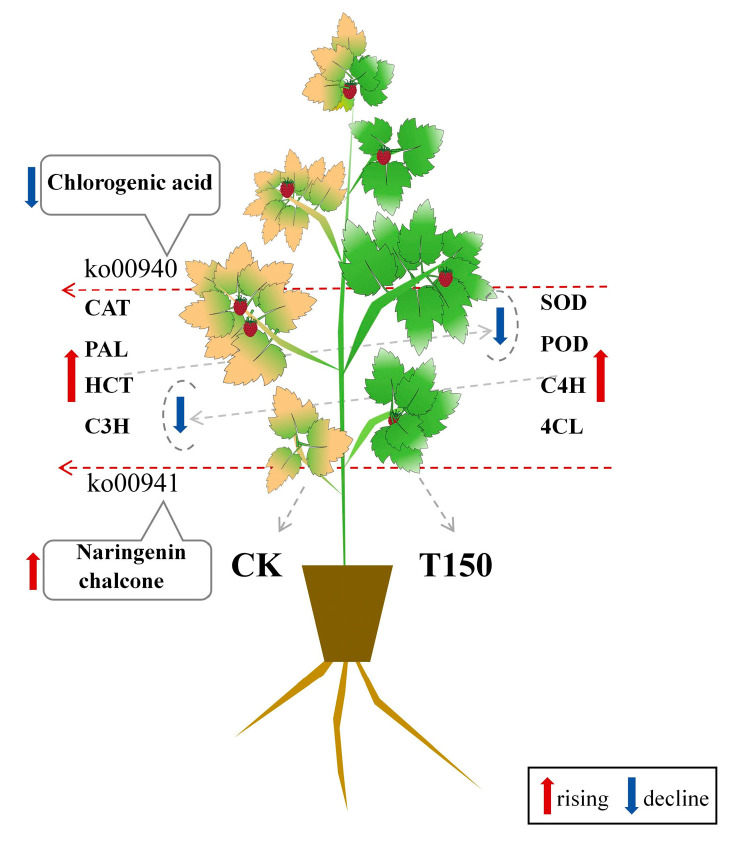
Joint transcriptome and metabolome results.

Naringenin chalcone belongs to the flavonoid class of phytochemicals and is found in the peel of red tomatoes. In a study investigating the effect of potassium humate on waterlogged barnyard grass, it was observed that potassium humate influences the flavonoid biosynthesis pathway. Specifically, p-coumaryl-CoA is converted to naringenin chalcone by chalcone synthase (CHS), which is then transformed into naringenin by chalcone isomerase (CHI). These flavonoid metabolites were significantly downregulated following the application of potassium humate [65]. Additionally, some studies have shown that the addition of naringenin chalcone to cancer cells induces chromatin condensation, DNA fragmentation, and vacuole formation [66], leading to the production of apoptotic bodies and autophagic vacuoles. Naringenin chalcone can inhibit cancer cell growth by inducing early and late apoptosis and promoting autophagy in animals. However, few studies have investigated its role in plants. It is therefore speculated that naringenin chalcone may have a similar function in plants. In this experiment, naringenin chalcone levels significantly increased in prematurely senescent raspberries. Its upregulation may trigger apoptosis in senescent cells and redirect nutrients to other organs, but it also inhibits the growth of new cells, ultimately causing the leaves to display signs of aging (Figure 14).

## 5. Conclusions

Compared with T150 plants, CK plants exhibited distinct differences in phenotype, physiology, and leaf structure: CK showed reduced plant height, stem diameter, bud number, and root activity, alongside increased root-to-crown ratio and root length. Additionally, CK had thicker lower leaves, while the upper and middle leaves displayed loosely arranged chloroplasts. The chlorophyll, soluble protein, and soluble sugar contents in CK leaves were lower than those in T150. In contrast, T150 plants showed higher SOD and POD activities in lower leaves, lower CAT activity, and significantly lower MDA and O_2_^−^ contents in lower leaves compared to CK.

Transcriptomic analysis identified 2062 upregulated and 2288 downregulated genes between the two types, with enrichment in pathways such as ribosome and photosynthesis. Specifically, T150 downregulated senescence-related genes while upregulating genes involved in chlorophyll metabolism and antioxidant enzyme synthesis. Metabolomic analysis detected 135 differential metabolites, mainly enriched in phenylpropanoid and flavonoid biosynthesis pathways: CK upregulated 19 triterpenoids, whereas T150 upregulated 15 flavonoids. Multi-omics integration highlighted the phenylpropanoid and flavonoid biosynthesis pathways as core regulatory hubs. T150 delays leaf senescence by maintaining flavonoid-mediated redox balance, while CK undergoes leaf senescence due to altered contents of chlorogenic acid and naringenin chalcone.

These findings link phenotypic plasticity to molecular reprogramming, demonstrating that adequate nitrogen supply delays leaf senescence through multiple mechanisms (e.g., maintaining photosynthetic efficiency, enhancing antioxidant capacity, regulating flavonoid metabolism). This underscores precision nitrogen management as a key strategy for modulating senescence in red raspberry leaves.

## Data Availability

Data are contained within the article and Supplementary Materials.

## Supplementary Materials

The following supporting information can be downloaded at: https://www.mdpi.com/article/10.3390/plants14152388/s1, Figure S1: Molecular Feature Analyses: Length Distributions, Species Classifications, and qRT-PCR Validations; Figure S2: Multivariate Statistical Analysis: PCA, OPLS-DA, and Permutation Validation Plots; Table S1: Phenotypic indices of red raspberries at the budding stage under three nitrogen application levels; Table S2: Morphological differences between normal and premature senescent red raspberries; Table S3: Contents of chlorophyll and carotenoids in leaves of normal and prematurely senescent raspberries; Table S4: Statistics of data filtering; Table S5: The top 20 metabolites identified in two phenotypic red raspberry groups under positive ion mode; Table S6: The top 20 metabolites identified in two phenotypic red raspberry groups under negative ion mode; Table S7: Common pathway information of differential metabolites and differentially expressed genes; Table S8: qRT-PCR Primer Information; Table S9: Data Tables Related to Figure 5 and Figure 6; Table S10: Data Tables Related to Figure 8, Figure 9, Figure 10, Figure 11, Figure 12 and Figure 13; Table S11: Data Tables Related to Figures S1 and S2.
