# Supplementary figures and images for: Integrated Transcriptomic and Metabolomic Analysis Reveals Nitrogen-Mediated Delay of Premature Leaf Senescence in Red Raspberry Leaves
Source: Plants (Basel). 2025 Aug 2;14(15):2388. doi: 10.3390/plants14152388 (PMC12349029; doi:10.3390/plants14152388)

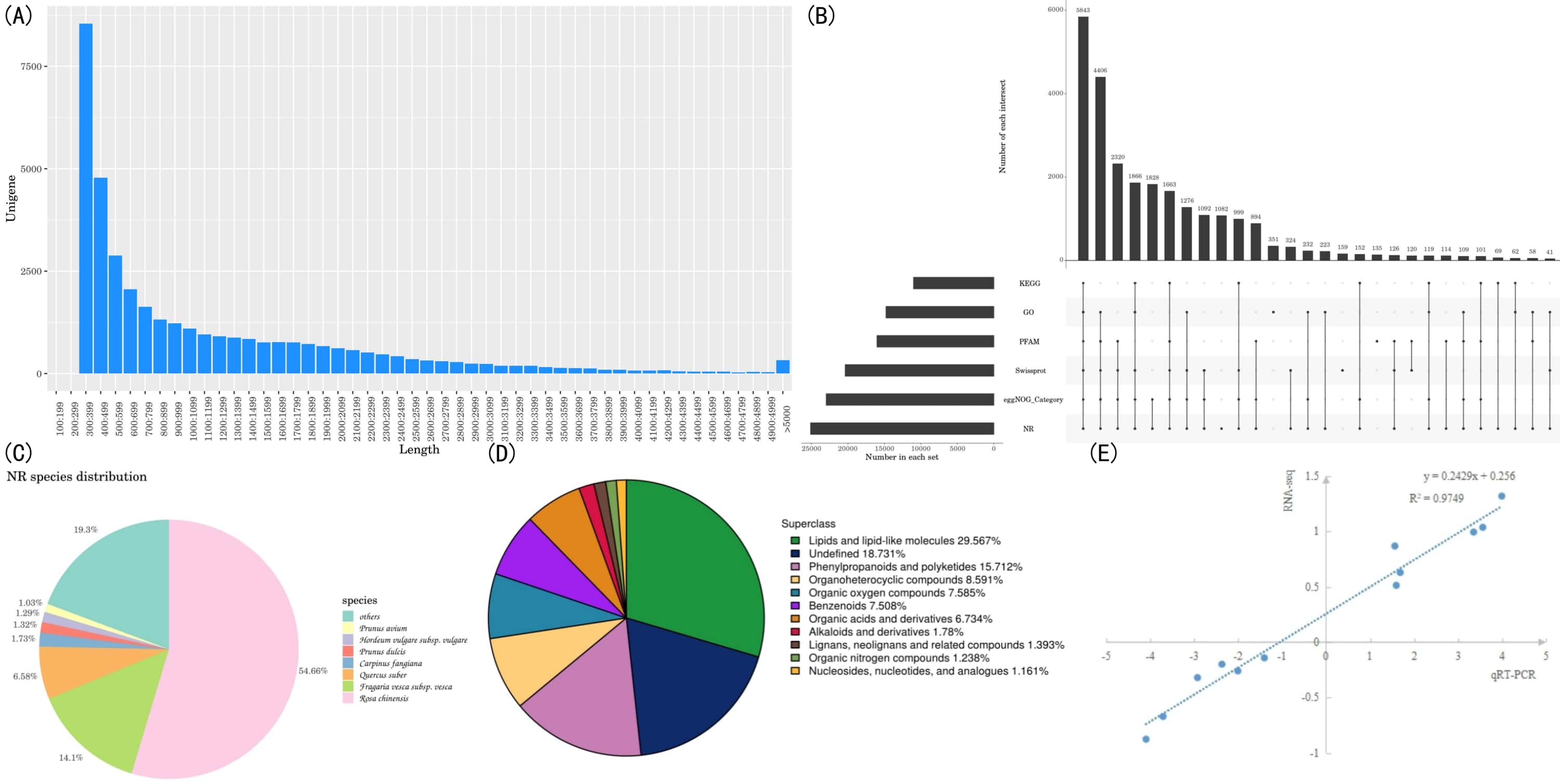

Supplement: Supplementary file 1 [file plants-14-02388-s001.zip › Supplementary Figure S1.jpg]

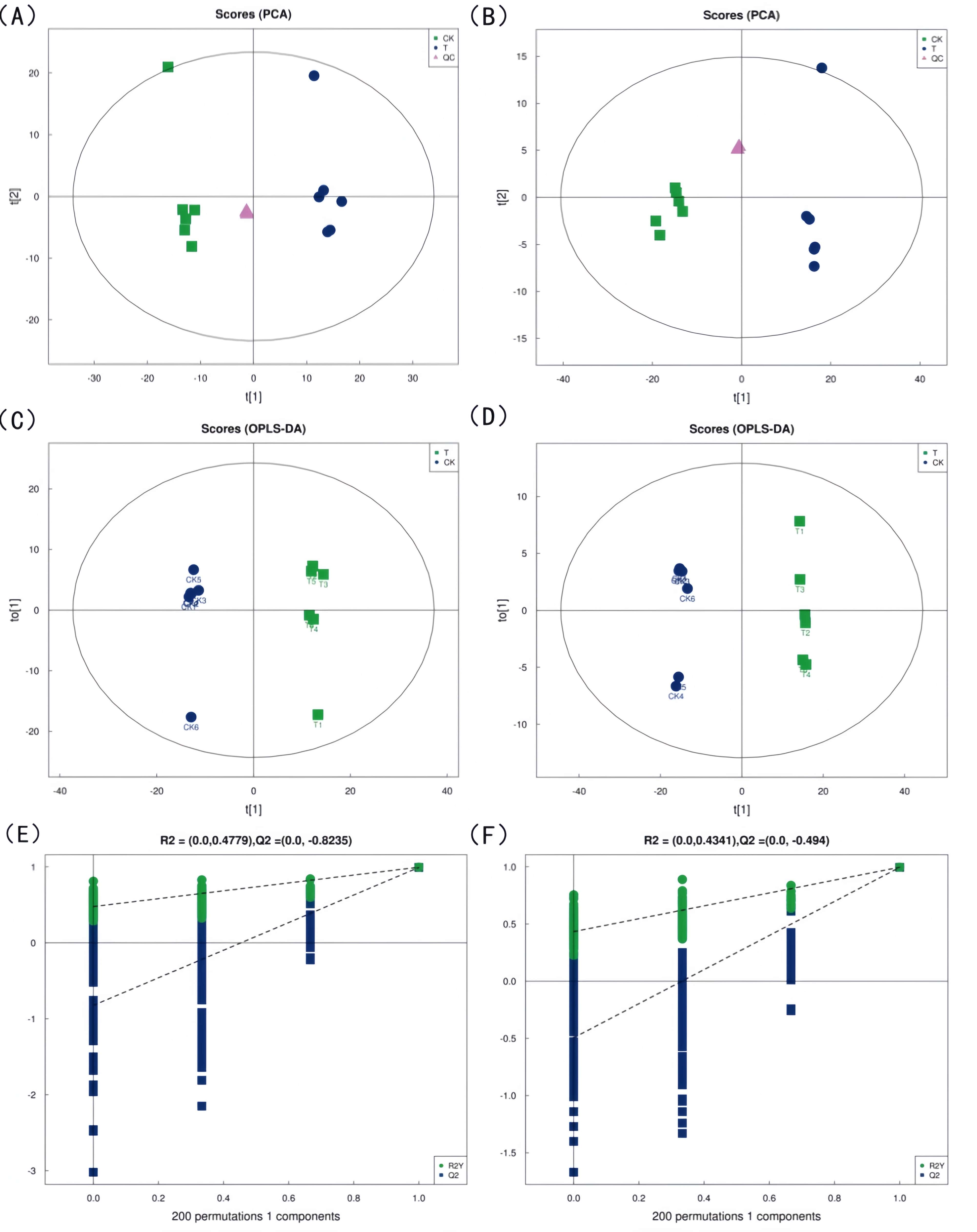

Supplement: Supplementary file 1 [file plants-14-02388-s001.zip › Supplementary Figure S2.jpg]
